# Post Irradiation Spindle Cell Carcinoma of Tonsillar Pillar

**DOI:** 10.1155/2011/325193

**Published:** 2011-11-30

**Authors:** Prateek Kinra, V. Srinivas, Kavita Sinha, Vibha Dutta

**Affiliations:** Department of Pathology, Armed Forces Medical College, Pune 411040, India

## Abstract

Spindle cell carcinoma of the tonsillar pillar is a rare malignancy. A case of spindle cell carcinoma of the anterior tonsillar pillar in a 59-year-old man is presented. A growth on the anterior tonsillar pillar, measuring 9 × 7 × 6 mm, was resected. The neoplasm occurred as a complication of radiotherapy (excessive cumulative radiation dose of 60 Gray) for carcinoma larynx with a latency period of three years. Postradiation spindle cell carcinoma is an uncommon disease manifesting as sarcoma in a previously irradiated field, usually with a latent period of 5 years or more. Literature is limited to small series. Histologically, this tonsillar growth was composed of a squamous cell carcinoma (epithelial component) and a spindle cell sarcomatous component. The two components of the tumour were confirmed using the immunohistochemical staining (cytokeratin and vimentin). Further p53 positivity of the sarcomatous elements aided in ruling out radiation-induced nonmalignant changes of mesenchymal tissue. This paper discusses this rare tumour in a common setting.

## 1. Introduction

Spindle cell carcinoma is an unusual tumour of the upper aerodigestive tract, characterized by squamous cell carcinoma and a sarcoma-like stroma that has intrigued surgical pathologists for years. Reports of more than 100 such cases in the literature reveal a constellation of characteristic findings [1]. It is more common in males in the sixth or seventh decade and presents as a tumour in the larynx, oral cavity, pharynx, or oesophagus—in that order of frequency. A history of radiation is common but not the rule [2]. The histological examination generally shows a squamous carcinoma in association with underlying, atypical, fusiform cells that often overshadow the carcinoma. Much debate has been devoted to the histogenesis of these tumours and especially to the nature of their bizarre stroma. The controversy is reflected in the different names given to the tumour: spindle cell carcinoma, carcinosarcoma, pseudosarcoma, sarcomatoid carcinoma, and polypoidal squamous cell carcinoma. We present a case of radiation-induced spindle cell carcinoma in the oral cavity.

## 2. Case History

A 56-year-old male presented to the surgery out patient department with cervical lymphadenopathy. The fine needle aspiration revealed a metastasis of squamous cell carcinoma. On indirect laryngoscopy, the patient was detected to have a growth in the larynx. Total laryngectomy and partial pharyngectomy with radical neck dissection were carried out. The histopathological examination of the tumour revealed a moderately differentiated squamous cell carcinoma wherein the margins were clear. Subsequently, the patient received 60 Gy radiotherapy for 30 sittings (2 Gy/fraction) over 2 weeks on the neck region. Postoperative period followup was uneventful although the patient developed hoarseness of voice. During a regular followup after a period of three years, the patient was detected to have a growth on the right anterior pillar of tonsillar fossa measuring 9 mm in greatest dimension. The growth was friable but did not bleed on touch. It was resected and sent for histopathological examination.

### 2.1. Histopathological Examination

**Figure 1:**
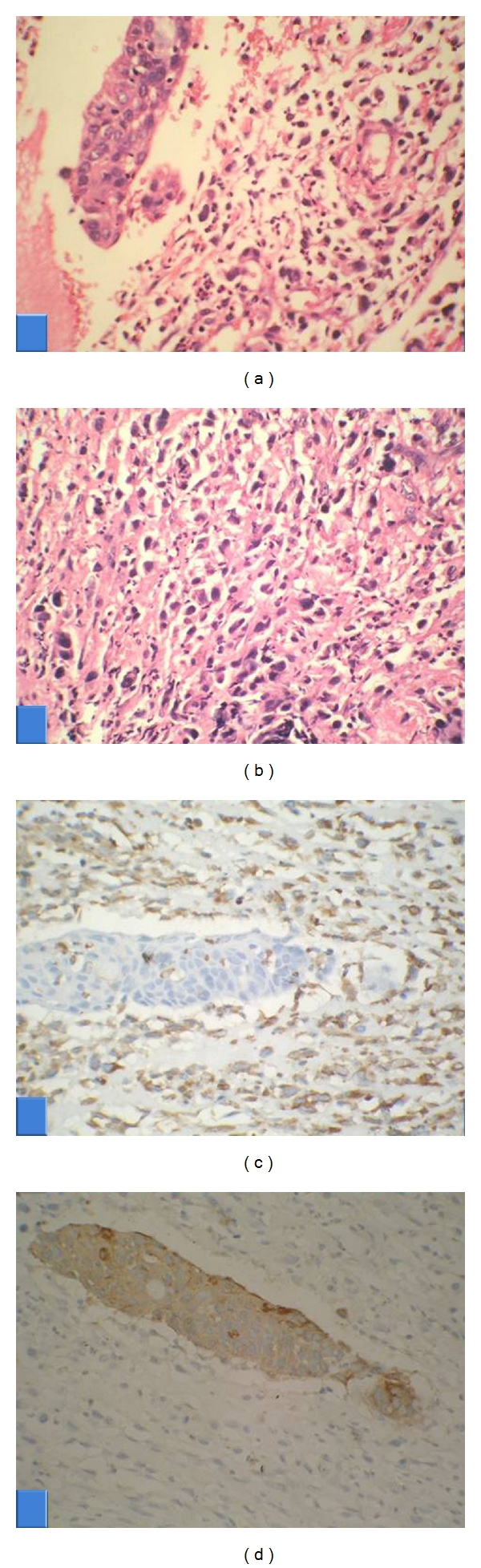
(a)–(d) Haematoxylin and eosin stained slides of tumour showing carcinomatous epithelial islands within sarcomatous elements ((a) 400x). Sarcomatous spindled cells with pleomorphic nuclei ((b) H&E, 400x). Immunohistochemistry: vimentin IHC showing diffuse positivity of spindled sarcoma cells ((c) 200x). Pan-cytokeratin IHC showing positivity of epithelial carcinoma cells ((d) 400x).

The biopsied tissue was processed and five-micron-thick sections were cut from routine formalin-fixed, paraffin-embedded tissue, and stained by hematoxylin and eosin stain for microscopic examination. Immunohistochemistry (IHC) was done using streptavidin-biotin immunoperoxidase technique (Envision Kit, M/s Dakopatts, Denmark) using monoclonal antibodies to pan-cytokeratin, vimentin, and polyclonal antibody for p53 (all antibodies were prediluted and obtained from M/s BioGenex, USA). Histologically, the resected mass showed a biphasic cell population. There were epithelial cell islands that were morphologically squamoid in appearance admixed with neoplastic spindle cell stroma consisting of bizarre nuclei and marked variation in cell size. Hyaline globules, bone, or cartilage elements were looked for in tumor areas but were not demonstrable (Figures 1(a) and 1(b)). Minimal necrosis was seen. Immunohistochemically, cytokeratin decorated the neoplastic epithelial cell nests and mesenchymal spindled tumour cells were observed to be vimentin positive, with sharp areas of demarcation. (Figures 1(c) and 1(d)). Based on these results, the tumor was labeled as a spindle cell carcinoma. The malignant stromal component also showed >50% nuclei positive for p53.

### 2.2. Followup

The patient was followed up for a period of three months, during which he was symptom-free.

## 3. Discussion

Spindle cell carcinoma is a rare biphasic malignant neoplasm which is composed of both malignant epithelial and malignant mesenchymal elements. Spindle cell carcinoma have been described in several organs, including uterus, bladder, lung, and others [3]. 

Radiation-induced malignancy is a well-known long-term complication of radiation therapy. Criteria for diagnosing malignancy as radiation induced were firmly established by Cahan et al. in 1948 [4]. These criteria comprised that (1) the patient had undergone radiation therapy, (2) the malignancy arose in the previously irradiated field, (3) histological evidence of a sarcoma, (4) latency period of at least five years between radiation and the presentation of the sarcoma and (5) primary and secondary tumor are of a different histological entity. With the technological advance in radiation therapy, Cahan's criteria were revised by Murray et al. in 1999 concerning the limitations on the latency period to as less as one year after radiation was included [5]. Even the index case discussed had a latency of three years. Data suggest that there is no relationship between cumulative radiation dose and the interval between exposure to radiotherapy and the development of radiation-induced sarcoma [6]. Mechanisms by which radiation may induce genetic changes leading to malignant transformation include loss of heterozygosity in directly radiated cell nuclei and by release of cytokines [7].

With an estimated incidence of 0.4 to 1.0%, radiation-induced malignancy is a rare entity. Radiation-associated sarcomas in particular are uncommon, constituting less than 5% of all sarcomas. Less than 1% of all radiation-induced sarcomas arise within the head and neck region and thus is a rare entity with few reported series in the international literature. Despite the low incidence of radiation-induced sarcoma, this pathological entity is today expected to be seen more frequently, due to an increased life expectancy and increased effectiveness of cancer therapy [6]. 

Histologically, the characteristic feature of the spindle cell carcinoma is the presence of malignant epithelial and heterologous stromal components. There are three hypotheses that can explain the histogenesis of spindle cell carcinoma: (1) multiclonal origin of the tumour arising from two or more stem cells (collision theory). This hypothesis is supported by the fact that electron microscopy of the cells reveals prominent amounts of endoplasmic reticulum, lysosomes, myelin fibers, and pseudopodia, indicating that the spindle cells are fibrotic and histiocytic rather than epithelial in origin; (2) monoclonal origin from a single totipotential stem cell that differentiates in separate epithelial and mesenchymal directions—hinting towards metaplastic carcinoma. This hypothesis is proved by the immunohistochemical and molecular analyses; (3) there is an atypical reactive proliferation of stroma as a response to the carcinomatous elements [8, 9].

In the case discussed, there is a sharp demarcation between the carcinomatous and sarcomatous elements without a transition zone, and the different immunohistochemical staining pattern of the two components for mesenchymal and epithelial markers, suggesting a different origin for each tumor cell type against the metaplastic carcinoma hypothesis. P53 positivity of sarcoma elements helped us in differentiating true sarcoma from radiation-induced nonmalignant changes of the mesenchymal tissue. However, possibility remains that the demarcation may not signify a different origin, since carcinoma components are demarcated in some sarcomatoid carcinomas in which the sarcomatous component derives from metaplasia of the epithelial cells. However, the positive immunohistochemical staining for vimentin in the sarcomatous elements of our case is not diagnostic of mesenchymal cells, as several types of carcinomas, including squamous cell carcinomas, show coexpression of vimentin and cytokeratin [10]. The differential diagnosis of the case discussed could include (i) inflammatory myofibroblastic tumour and (ii) high-grade myofibroblastic sarcoma. 

The findings of this tumour on computerized tomography (CT) are not specific and can be similar to other neoplasms. CT may reveal malignant features such as ill-defined margins, calcification, irregular rim enhancement, and calcification [11]. The primary treatment for spindle cell carcinoma is same as for squamous cell carcinoma, and surgical excision is the preferred treatment [11]. In the present case, the tumor was excised with an adequate margin. Poor prognosis has been reported in patients treated with radiotherapy, which is considered to be ineffective although adjuvant irradiation may be beneficial in patients who have positive surgical margins or who have nodal metastasis at the time of diagnosis [12]. The role of chemotherapy has not been established, but it may decrease the incidence of recurrence or metastasis of primarily sarcomatous tissue. The lethality for oral spindle cell carcinoma has been reported to be 60%. The mean survival time of the patients who died of this tumour was 1.9 years [1]. 

The presented case demonstrates that clinicians managing cancer patients as well as pathologists have to be aware of radiation-induced spindle cell carcinoma as a long-term complication of treatment, especially as this pathological entity is today expected to be seen more frequently due to an increased life expectancy combined with improved survival of cancer patients resulting from an increased effectiveness of cancer therapy. As the prognosis of radiation-induced malignancies in general is poor and the treatment options are limited, early diagnosis and a correspondingly better chance of complete surgical excision assume great importance.
